# Genotype-Specific Recruitment of Rhizosphere Bacteria From Sandy Loam Soil for Growth Promotion of *Cucumis sativus* var. *hardwickii*

**DOI:** 10.3389/fmicb.2022.910644

**Published:** 2022-06-27

**Authors:** Zeyang Li, Yingying Zheng, Yansu Li, Xu Cheng, Sanwen Huang, Xueyong Yang, Yuxuan Qin

**Affiliations:** ^1^School of Agricultural Sciences, Zhengzhou University, Zhengzhou, China; ^2^Key Laboratory of Biology and Genetic Improvement of Horticultural Crops of the Ministry of Agriculture, Sino-Dutch Joint Laboratory of Horticultural Genomics, Institute of Vegetables and Flowers, Chinese Academy of Agricultural Sciences, Beijing, China; ^3^Shenzhen Branch, Guangdong Laboratory of Lingnan Modern Agriculture, Genome Analysis Laboratory of the Ministry of Agriculture and Rural Affairs, Agricultural Genomics Institute at Shenzhen, Chinese Academy of Agricultural Sciences, Shenzhen, China

**Keywords:** rhizosphere bacteria, cucumber, growth promotion, genotype, plant-bacteria interaction

## Abstract

The composition and structure of the rhizosphere microbiome is affected by many factors, including soil type, genotype, and cultivation time of the plant. However, the interaction mechanisms among these factors are largely unclear. We use culture-independent 16S rRNA amplicon sequencing to investigate the rhizosphere bacterial composition and the structure of cultivated cucumber Xintaimici (XT) and wild-type cucumber *Cucumis sativus* var. *hardwickii* (HD) in four kinds of soils. We found that soil type, cultivation time, and genotype affected the composition and structure of cucumber rhizosphere bacterial communities. Notably, HD showed better physiological features in sandy soil and sandy loam soil than it did in black soil and farm soil at 50 days post-sowing, which was due to its stronger recruitment ability to *Nitrospira*, *Nocardioides*, *Bacillus*, and *Gaiella* in sandy soil, and more *Tumebacillus*, *Nitrospira*, and *Paenibacillus* in sandy loam soil. Meanwhile, we also found that HD showed a better recruiting capacity for these bacterial genera than XT in both sandy soil and sandy loam soil. Functional predictions indicated that these bacteria might have had stronger root colonization ability and then promoted the growth of cucumbers by enhancing nitrogen metabolism and active metabolite secretion. In this study, our findings provided a better insight into the relationship between cucumber phenotype, genotype, and the rhizosphere bacterial community, which will offer valuable theoretical references for rhizosphere microbiota studies and its future application in agriculture.

## Introduction

The rhizosphere refers to a narrow zone surrounding plant roots in which chemical and biological features of the soil are affected by the structure and exudates of the roots (Saleem et al., 2018; Vives-Peris et al., 2020). The interaction of plants and microorganisms is highly active in the rhizosphere, which provides a better survival environment for microorganisms (Berendsen et al., 2012). Rhizosphere microorganisms play important roles in plant growth and development by influencing plant nutrient absorption efficiency, responding to biotic and abiotic stresses, and secreting other metabolites (Trivedi et al., 2020).

The composition and structure of plant rhizosphere microbial communities are affected by a variety of factors, including soil types, plant genotypes, and cultivation times (Wang et al., 2017). This study showed that the microbial community and the abundance of microorganisms in the rhizosphere of *Arabidopsis thaliana* were strongly affected by soil types (Lundberg et al., 2012). In rice, the difference between microbial communities recruited by *indica* and *japonica* in the same soil contributed to the utilization efficiency of nitrogen (Zhang J. et al., 2019). In sorghum, there was a significant difference in the bacterial composition of the rhizosphere of the same soil at 10 and 50 days after sowing (Schlemper et al., 2017). In addition, the rhizosphere microbial communities of the sweet potato (Marques et al., 2014) and lettuce (Schreiter et al., 2014) were also affected to varying degrees by the above-mentioned factors. Although the evaluation of these factors has been addressed individually, whether the environment, genotype, and cultivation time could interact and influence the composition and structure of the rhizosphere microbiome is still not well-understood.

Cucumber (*Cucumis sativus* L.) is one of the most important vegetables in the world, with extremely high economic value (Feng et al., 2020). According to the Food and Agriculture Organization of the United Nations (FAO),^1^ in 2019, the cultivation area of cucumbers in China had reached 1,258,370 ha, with a total production of 70,338,971 tons. Excessive usage of chemical fertilizers is a common phenomenon in cucumber cultivation. The overuse of chemical fertilizers inevitably leads to a series of problems, such as the decline of cucumber quality, causing environmental pollution, and reducing soil sustainability. Therefore, safe, effective, and environmentally friendly green fertilizers are urgently needed (Haskett et al., 2021). In recent years, plant growth-promoting rhizobacteria (PGPR) have been widely used as bio-fertilizers. Studies have shown that PGPR can promote the nutrient utilization efficiency of plants, increase plant yield, and reduce the use of traditional chemical fertilizers (Ijaz et al., 2019).

Although numerous papers have reported the effects of factors, such as soil, genotype, and cultivation time, on the composition of plant rhizosphere bacterial communities, few studies focus on the comprehensive impact of these factors on rhizosphere bacterial communities. Thus, in this study, culture-independent 16S rRNA amplicon sequencing and performed experience were used to investigate the impacts of soil type, genotype, and cultivation time on the composition and dynamics of the rhizosphere bacterial structure. Meanwhile, the growth promotion mechanisms of specific rhizosphere bacteria to cucumber were investigated. The result of the study will provide theoretical references for using synthetic bacterial communities to promote the growth of cucumbers and other horticultural crops in the future.

## Materials and Methods

### Plant Materials and Soil

The seeds of cultivated cucumber Xintaimici (XT) and wild-type cucumber *C*. *sativus* var. *hardwickii* (HD) were provided by the Functional Gene Research Group of the Institute of Vegetables and Flowers, Chinese Academy of Agricultural Sciences. We collected four kinds of soil from three different cities in China. Sandy and sandy loam soils were collected from Shouguang City, Shandong Province, China (118.791°E, 36.855°N). Black soil was collected from Linghai City, Liaoning Province, China (121.309°E, 41.087°N), and farm soil was collected from Beijing, China (116.333°E, 39.962°N).

### Plant Germination, Transplant, and Cultivation in the Greenhouse

The seeds of XT and HD were surface sterilized in 75% ethanol for 15 s and 0.3% sodium hypochlorite for 15 min, then washed six times with sterile distilled water. The seeds were germinated on a sterile paper and incubated at 28°C in the dark until the roots reached 1 cm (24 h). After germination, the cucumber seedlings were transplanted into pots with four different kinds of soils and grown in the greenhouse of the Southern District of the Institute of Vegetables and Flowers, Chinese Academy of Agricultural Sciences, China (116.333°E, 39.962°N) under the conditions of 16/8 h light/dark and 28°C/18°C day/night. The pots were watered every 2 days with sterilized water and sterilized with modified 1/4 Hoagland’s nutrient solution [1X Hoagland’s nutrient solution: MgSO_4_ 1 mM, CaCl_2_ 4 mM, KNO_3_ 10 mM, NH_4_H_2_PO4 1 mM, KCL 10 mM, Ca(H_2_PO_4_) ⋅ 2H_2_O] 0.5 mM and trace element powder purchased from Coolabern 74.93 mg/L) every 4 days. No chemical fertilizers or pesticides were used in this experiment.

### Measurement of Plant Physiological Features

Physiological features were measured at 30 days post-sowing (30 DPS) and 50 days post-sowing (50 DPS) of XT and HD in all four kinds of soil. Specifically, plant height was measured from the base of the shoot to the tip of the tallest leaf; the stem diameter was measured with a caliper just below the cotyledon scar; and the leaf area was calculated with this formula: leaf area = leaf length (leaf base notch to leaf tip) × leaf width (the widest part of the leaf) × 0.87. At each time point, for each soil type and cucumber genotype, triplicate samples were taken.

### Collection of the Samples

Rhizosphere soil samples of XT and HD in four kinds of soils were collected at 30 and 50 DPS. At each time point, for each soil type and cucumber genotype, triplicate samples were collected. Roots with tightly attached rhizosphere soil were placed in a sterile tube with 25 ml of sterile water and vortexed for 30 s. Then, we transferred the supernatant to another new tube and centrifuged it for 10 min at 10,000 rpm. Following that, the supernatant was discarded, and the sample was collected from the bottom of the tube and then stored at −80°C before DNA extraction.

### Sterilized and Non-sterile Treatment of Sandy Loam Soil

The sterilized soil of sandy loam soil was obtained by autoclaving it three times for 30 min. After germination, both XT and HD seedlings were transplanted into pots containing sterilized and non-sterilized sandy loam soil, respectively, and grown in a greenhouse. Physiological features were measured at 40 days post-sowing (40 DPS). The triplicate samples were taken per treatment.

### DNA Extraction, PCR Amplification, and Sequencing

DNA was extracted from 0.25 g of each soil sample using the DNeasy PowerSoil Pro Kit (QIAGEN HB-2485) by following the manufacturer’s instructions. The concentration and quality of the DNA were evaluated by the NanoDrop 2000 spectrophotometer (Thermo Fisher Scientific, MA, United States). The V3–V4 region of the 16S rRNA gene was amplified using the universal primers (341F: 5′-CCTACGGGNBGCASCAG-3′ and 806R: 5′-GACTACNVGGGTATCTAATCC-3′) (Sun et al., 2013). Every 50 μl PCR reaction contained 2 μl of diluted DNA, 25 μl of 2X Phanta Max Master Mix (Vazyme P515-01, China), 10 pM of forward and reverse primers, and 21 μl of Nuclease-Free Water (PROMEGA). The thermal cycling consisted of initial denaturation at 95°C for 30 s, followed by 28 cycles of 95°C for 15 s, 55°C for 15 s and, 72°C for 30 s, finally at 72°C for 5 min. The PCR products were purified using the QIA quick Gel Extraction Kit (QIAGEN Germany). The sequencing library was generated using the KAPA Library Preparation Kit (Kapa, MA, United States) by following the manufacturer’s instructions and was quantified by the Agilent Bioanalyzer 2100 system. Finally, the quantified library was sequenced on the Novaseq PE250 platform (Illumina, CA, United States), with the generation of 2 × 250 base pairs.

### Bioinformatics Analysis of 16S rRNA Sequencing Data

The pair-end raw sequencing data were processed in the QIIME2 pipeline (Bolyen et al., 2019). Specifically, the q2-dada2 (Callahan et al., 2016) plugin was used to generate clean data. After that, taxonomy was assigned *via* the q2−feature−classifier (Bokulich et al., 2018; Robeson et al., 2020), and then, the mitochondrial and non-bacterial ASVs were removed. The diversity matrix was estimated and calculated by the q2-diversity plugin, and the phylogeny rooted tree used in the analysis was aligned and constructed by the q2-phylogeny plugin (Katoh et al., 2002; Price et al., 2010). To mitigate the impact of the inconsistent sequences, all samples were rarefied to 30,000 sequences for analysis. The Principal Coordinate Analysis (PCoA) plot was performed by the R Package vegan (v.2.5.7) (Beals, 1984), and a heat map was generated by the ggplot2 (v.3.3.5) package (Gómez-Rubio, 2017). The functional potential of a bacterial community was predicted using PICRUSt (v. 2.4.1) (Caicedo et al., 2020), and the deferentially enriched KOs were identified by a 1-tail Wilcoxon rank sum test. Further, a reporter score of *z* ≥ 1.64 (Patil and Nielsen, 2005) was used as a significant difference hold for differentiating KEGG pathways (Feng et al., 2015). We then visualized all the results by the ggplot2 package (v.3.3.5).

### Statistical Analysis

Statistical analysis was performed with SPSS software (V26.0.0.2). One-way ANOVA and two-tailed Student’s *t*-test were used for significance analysis between different samples.

## Results

### Physiological Features of Cucumber in Various Kinds of Soils

**FIGURE 1:**
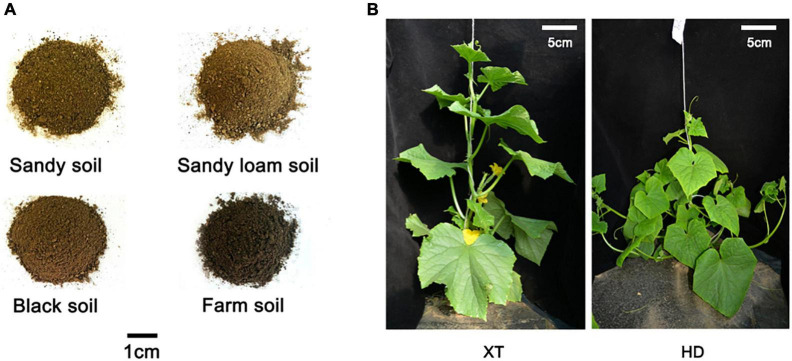
Four different soils and two different cucumber genotypes. **(A)** There are four kinds of representative soils selected from different regions: sandy soil, sandy loam soil, black soil, and farm soil. **(B)** XT on the left and HD on the right.

To explore the phenotype variation of cucumbers with distinctive genotypes in different soils, we cultivated two types of cucumbers, cultivated cucumber XT and wild-type cucumber HD, on four different kinds of soil, including sandy soil, sandy loam soil, black soil, and farm soil (Figure 1).

**FIGURE 2:**
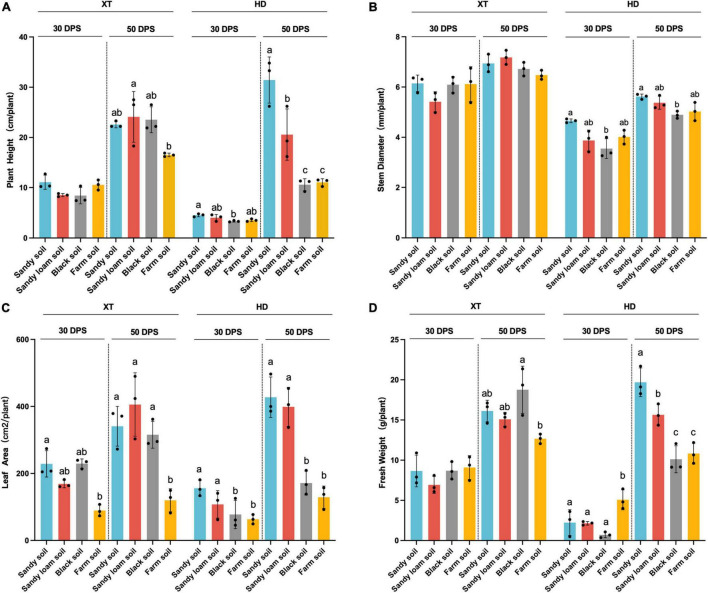
Statistical analysis of physiological characteristics. **(A)** Plant height of XT and HD in four different soils. **(B)** Stem diameter of XT and HD in four different soils. **(C)** Leaf area of XT and HD in four different soils. **(D)** Fresh weight of XT and HD in four different soils. Statistics recorded data of 30 and 50 DPS. Significances are indicated among different groups by *P*-values (ANOVA, Turkey HSD). Data are the means of three replicates, and error bars indicate standard deviations. Different lowercase letters indicate a significant difference (*p* < 0.05) in Tukey’s test.

We found that compared with 30 DPS, in sandy soil and sandy loam soil, at 50 DPS, the improvement of plant height, leaf area, and fresh weight of HD was more than twice that of XT (Figure 2). At the same time, we found that in sandy soil and sandy loam soil at 50 DPS, the plant height, leaf area, and fresh weight of HD were significantly enhanced, compared with in black soil and farm soil (*p* < 0.05). The average height and leaf area of HD in sandy soil increased by 197.48 and 149.45% compared to that in black soil and 183.89 and 231.27% compared to that in farm soil, respectively. The average leaf area in sandy loam soil increased by 132.72% compared with black soil and 209.05% compared with farm soil, respectively (Figure 2). Our results suggested that HD grows faster in sandy soil and sandy loam soil than in XT, and sandy soil and sandy loam soil were more conducive to HD growth than black soil and farm soil.

### Distinct Rhizosphere Bacterial Communities of Cucumber in Different Kinds of Soils

We have found that the growth states of HD in sandy soil and sandy loam soil were better than that of XT. We further explored the rhizosphere bacterial compositions of cucumbers grown in different soils at different cultivation times by 16S rRNA amplicon sequencing. We obtained 314,781 reads and 99,207 filtered amplicon sequence variations (ASVs) per sample on average from 72 samples (Supplementary Table 1).

**FIGURE 3:**
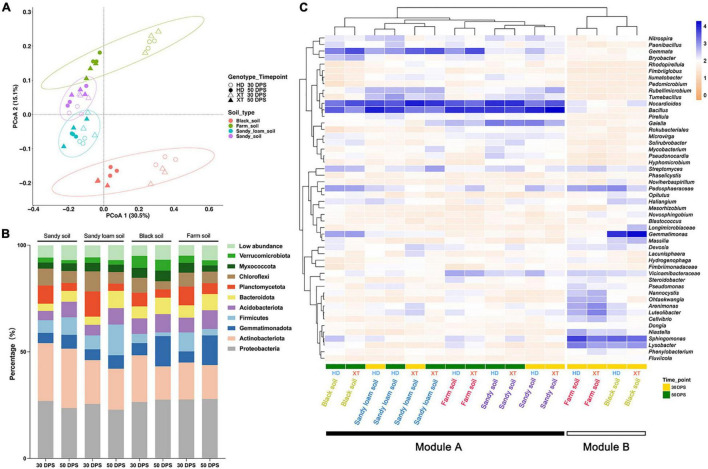
Differences in the composition of bacterial communities in different soils, cultivation times and genotypes. **(A)** PCoA (for principal coordinates PCo1 and PCo2) with Bray–Curtis distance shows that the different soils are separated in the area on the abscissa. Different samples assemble four circles regularly. The hollow circle represents HD at 30 DPS; the Solid circle represents HD at 50 DPS; the hollow triangle represents XT at 30 DPS; the solid triangle represents XT at 50 DPS. Purple is sandy soil; blue is sandy loam soil; green is farm soil; red is black soil. **(B)** The percentage of the bacterial community on the phylum level in different soils at different cultivation times. Different colors represent different bacterial phyla. **(C)** A heat map and hierarchical clustering show the average relative abundance of the top 50 genera of all samples. Yellow represents 30 DPS, and the green represents 50 DPS.

Principal coordinate analysis (PCoA) of Bray–Curtis distance was performed to reveal the overall dissimilarity of rhizosphere bacterial composition. The result showed that four kinds of soils were distributed into four clusters, which were separated along the second coordinate axis (Figure 3A). We found that the bacterial compositions of sandy soil and sandy loam soil were similar, whereas those of black soil and farm soil showed varied. Meanwhile, we found that black and farm soil samples collected at different time points had diverse bacterial communities (Figure 3A). Interestingly, we found that the clusters of rhizosphere bacteria in sandy soil and sandy loam soil at 30 and 50 DPS showed a high level of similarity. However, we did not observe the semblable phenomenon in black and farm soil. At the same time, in sandy soil, sandy loam soil and farm soil, we found no significant differences between the rhizosphere bacterial communities of the two genotypes (Figure 3A).

According to the sequencing results, over 90% of ASVs can be assigned to the phylum level on average (Supplementary Table 1). For all samples, the top three relatively abundant phyla were found to be Proteobacteria, Actinobacteria, and Gemmatimonadota, accounting for 28.9, 17.9, and 7.6%, respectively (Figure 3B and Supplementary Table 2). The abundance of Planctomycetota and Chloroflexi decreased at 50 DPS when compared to that at 30 DPS; they might be needed for the early development of XT and HD. The opposite trend, however, was found in Gemmatimonadota. At 50 DPS, we found the relative abundance of Firmicutes in sandy soil and sandy loam soil was as high as 8.2 and 14.4%, respectively, compared to that in black soil and farm soil, which only accounted for 1.7 and 3.0% (Figure 3B and Supplementary Table 2). Nevertheless, the relative abundance of Gemmatimonadota in black soil (14.2%) and farm soil (13.9%) was higher than that in sandy soil (6.5%) and sandy loam soil (6.3%) (Figure 3B and Supplementary Table 2). Our results showed that cucumbers can autonomously select their rhizosphere bacterial communities according to development time and soil environment.

At the genus level, the top 50 genera of all samples were selected for further investigation of cucumber rhizosphere bacterial composition (Supplementary Table 3); we generated a heatmap (Figure 3C) of these genera and identified two modules, including Modules A (the bacterial community of the rhizosphere in all kinds of soils at 50 DPS, as well as in sandy soil and sandy loam soil at 30 DPS) and B (the bacterial community of the rhizosphere in farm soil and black soil at 30 DPS). In Module A, there are more abundant *Nocardioides*, *Bacillus*, and *Gemmata*, which may play an important role in cucumber growth; the abundance of *Gaiella* in sandy soil is higher than in other soils, and there are more *Rubellimicrobium* and *Tumebacillus* in sandy soil and sandy loam soil than in black and farm soil. For black soil, it contained more *Pedospheraceae*, *Streptomyces*, *Gemmatimonas*, and *Bryobacter* than sandy soil, sandy loam soil, and farm soil. Module B contained more *Nannocystis*, *Ohtaekwangia*, *Arenimonas*, *Lureolibacter*, and *Cellvibrio* in farm soil than in black soil at 30 DPS; conversely, there were more *Massilia*, *Gemmatimonas*, and *Flavisolibacter* in black soil than in farm soil. The relative abundance of *Spingomonas* and *Lysobacter* in Module B is greater than in Module A; these genera may help cucumber development at an early stage in black soil and farm soil.

### Growth Promotion of *Hardwickii* Through Specific Rhizosphere Bacteria Recruitment From Sandy Soil and Sandy Loam Soil

**FIGURE 4:**
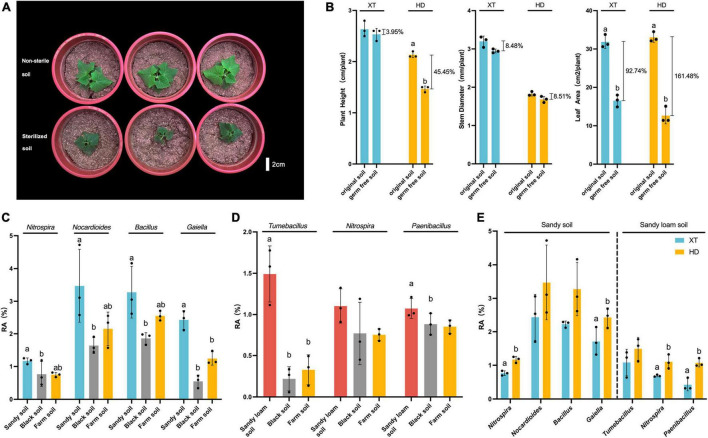
The dominant bacteria in HD rhizosphere of sandy soil and sandy loam soil. **(A)** HD planted in non-sterile soil and sterilized soil of sandy loam soil at 40 DPS. **(B)** Plant height, stem diameter and leaf area of XT and HD in non-sterile soil and sterilized soil of sandy loam soil at 40 DPS. **(C)** Relative abundances of *Nitrospira*, *Nocardioides*, *Bacillus* and *Gaiella* in three kinds of soils at 50 DPS. **(D)** Relative abundances of *Tumebacillus*, *Nitrospira*, and *Paenibacillus* in three kinds of soils at 50 DPS. **(E)** Relative abundances of *Nitrospira*, *Nocardioides*, *Bacillus* and *Gaiella* of XT and HD rhizosphere in sandy soil; relative abundances of *Tumebacillus*, *Nitrospira*, and *Paenibacillus* of XT and HD rhizosphere in sandy loam soil. Data are the means of three replicates, and error bars indicate standard deviations (*p* < 0.05); significance among different groups is indicated by Student’s *t*-test. Letters represent the results of Student’s *t*-test.

Since we found that HD grown in sandy soil and sandy loam soil was significantly better than that in black soil, farm soil, and Bray–Curtis distance suggested that sandy soil and sandy loam soil had similar bacterial communities (Figure 3A), we, therefore, speculated that the rhizosphere bacterial community in sandy soil and sandy loam soil contributed to the enhanced physiological features of HD. Then, we chose sandy loam soil to explore the effect of microorganisms on cucumber growth. We planted XT and HD on non-sterile and sterilized sandy loam soil to observe their growth states. We found a strong growth-promoting effect on HD by microorganisms in sandy loam soil (Figure 4A), and then, we measured plant height, stem diameter, and leaf area of XT and HD in both non-sterile soil and sterilized soil. For XT, we found that plant height, stem diameter, and leaf area of non-sterile soil increased by 3.95, 8.48, and 92.74% more than in sterilized soil, respectively; for HD, the height and leaf area were significantly higher in non-sterile soil compared to sterilized soil; they increased by 45.50 and 161.10%, respectively, and the stem diameter increased by 8.51% (Figure 4B). Therefore, we concluded that the microorganisms in sandy loam soil could promote the growth of cucumbers, and this promoting effect on HD was stronger than that of XT.

To further explore which bacteria may promote the growth of HD in sandy soil and sandy loam soil, we chose the top 20 genera (Top 20) (Supplementary Table 4) and the top 50 ASVs of all samples at 50 DPS to identify the dominant rhizosphere bacteria of HD in sandy soil and sandy loam soil.

For the rhizosphere bacteria of HD in sandy soil, we found the relative abundance of *Nocardioides*, *Bacillus*, *Rubellimicrobium*, *Gaiella*, *Tumebacillus*, and *Nitrospira* in sandy soil was higher than that in black soil, and farm soil (Supplementary Figure 1); *Nocardioides*, *Bacillus*, *Streptomyce*, *Gaiella*, and *Nitrospira* showed higher relative abundance at 50 DPS compared to 30 DPS (Supplementary Figure 2). Meanwhile, at 50 DPS, *Nocardioides*, *Bacillus*, *Vicinamibacteraceae*, *Gaiella*, *Nitrospira*, *and Paenibacillus* were more abundant compared to XT (Supplementary Figure 3). Finally, we got four dominant genera: *Nitrospira*, *Nocardioides*, *Gaiella*, and *Bacillus*. The relative abundance of *Nitrospira*, *Nocardioides*, *Bacillus*, and *Gaiella* in the rhizosphere of HD in sandy soil was significantly higher than that in black soil (Figure 4C). Meanwhile, the relative abundance of *Nitrospira* and *Gaiella* in the rhizosphere of HD, accounting for 1.17 ± 0.07% and 2.43 ± 0.24%, respectively, were significantly higher than that of XT, accounting for 0.77 ± 0.06% and 1.71 ± 0.34% (Figure 4E).

For the rhizosphere bacteria of HD in sandy loam soil, we found that the abundance of *Rubellimicrobium*, *Tumebacillus*, *Nitrospira*, *and Paenibacillus* in HD rhizosphere were higher at 50 DPS in sandy loam soil than that in black soil and farm soil (Supplementary Figure 1); bacterial genera of *Luteolibacter*, *Gemmata*, *Tumebacillus*, *Nitrospira*, and *Paenibacillus* were enriched at 50 DPS compared to that at 30 DPS (Supplementary Figure 4). Meanwhile, *Bacillus*, *Luteolibacter*, *Pedosphaeraceae*, *Tumebacillus*, *Nitrospira*, *and Paenibacillus* showed higher relative abundance at 50 DPS compared to XT during the same period (Supplementary Figure 5). Therefore, we believed that HD grown in sandy loam soil favorably recruited *Tumebacillus*, *Nitrospira*, *and Paenibacillus* (Figure 4D). To be more specific, at 50 DPS in sandy loam soil, the relative abundance of *Tumebacillus* (1.49 ± 0.28%) in the rhizosphere of HD was more than four times higher than that in black soil (0.22 ± 0.12%) and farm soil (0.33 ± 0.15%) (Figure 4D); the relative abundance of *Nitrospira and Paenibacillus* in the rhizosphere of HD were 1.10 ± 0.17% and 1.07 ± 0.10%, which were significantly higher than that of XT, accounting for 0.69 ± 0.02% and 0.43 ± 0.15% (Figure 4E).

**FIGURE 5:**
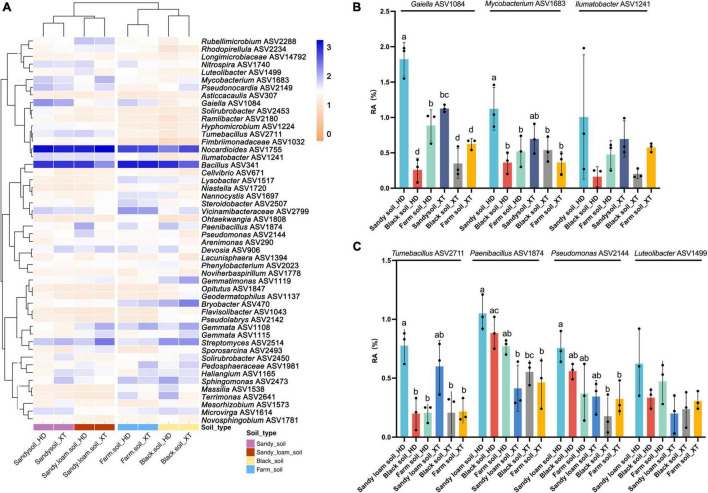
Top 50 ASVs of 50 DPS in all samples. **(A)** A heat map and hierarchical clustering show the average relative abundance of the top 50 ASVs of 50 DPS samples. **(B)** Relative abundances of *Gaiella* ASV1084, *Mycobacterium* ASV1683, and *Ilumatobacter* ASV1241 in XT and HD in sandy soil, black soil and farm soil at 50 DPS. **(C)** Relative abundances of *Tumebacillus* ASV2711, *Paenibacillus* ASV1874, *Pseudomonas* ASV2144, and *Luteolibacter* ASV1499 of XT and HD in sandy loam soil, black soil and farm soil at 50 DPS. Data are the means of three replicates, and error bars indicate standard deviations (*p* < 0.05); significance among different groups is indicated by *P*-values (ANOVA, Turkey HSD). Letters represent the results of Tukey’s test.

In addition to the genus-level findings, from the heat map of ASV level, we found that the rhizosphere bacterial ASVs in sandy soil and sandy loam soil were clustered together, as were those in black soil and farm soil. Besides, all 50 DPS samples contained significant amounts of *Nocardioides* ASV1755 and *Bacillus* ASV341 (Figure 5A). In sandy soil, the relative abundance of *Gaiella* ASV1084 and *Mycobacterium* ASV1683 in HD was significantly higher than that in black soil and farm soil, with an average proportion as high as 1.82 and 1.13%, respectively. *Ilumatobacter* ASV1241 (0.77%) showed the same tendency. At the same time, HD recruits more of these ASVs than XT in sandy soil (Figures 5A,B). In sandy loam soil, HD rhizosphere contained more *Tumebacillus* ASV2711 (0.77%), *Paenibacillus* ASV1874 (1.05%), *Pseudomonas* ASV2144 (0.76%), and *Luteolibacter* ASV1499 (0.62%) than in black soil and farm soil; the relative abundances of these rhizosphere bacterial ASVs of HD were higher than that of XT (Figures 5A,C). Our results finally proved that HD showed a stronger recruitment capability for *Nocardioides*, *Bacillus*, *Gaiella*, *Nitrospira*, *Tumebacillus*, and *Paenibacillus*. In addition, ASVs from these genera, such as ASV1755, ASV341, ASV1084, ASV2711, and ASV1874, may play a leading role in bacteria-promoting HD growth. ASVs from other different genera, such as *Mycobacterium* ASV1683, *Ilumatobacter* ASV1241, *Pseudomonas* ASV2144, and *Luteolibacter* ASV1499, were also strongly recruited by HD. All these bacteria may play a crucial role in promoting HD growth in sandy soil and sandy loam soil.

### Functional Prediction of Rhizosphere Bacterial Community

**FIGURE 6:**
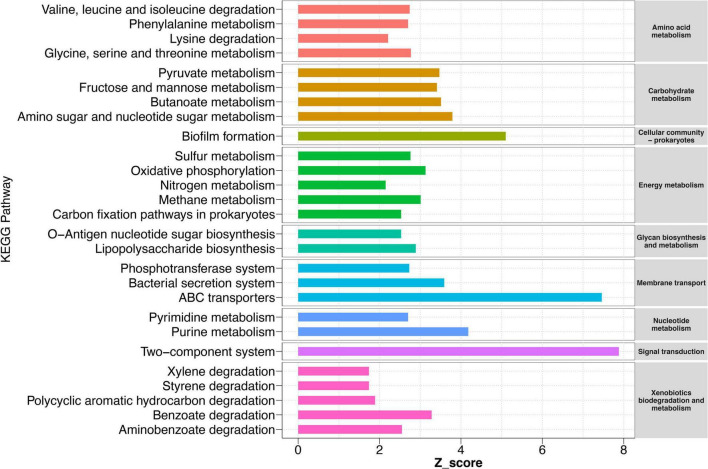
KEGG pathways of rhizosphere bacteria sample in different soils. Pathways are enriched in sandy and loam soil compared to black soil and farm soil. Pathways with a significant difference in reporter score (>1.64, enriched in sandy soil and sandy loam soil) were retained. Reporter scores > 1.64 are shown on the map. Different colors represent different metabolic pathways of KEGG level 2 (right) and level 3 (left).

Although our results have indicated that HD had a stronger ability to recruit certain bacteria in sandy soil and sandy loam soil, what functions these bacteria perform and the potential functions of other bacteria for cucumber remained largely unknown. Therefore, we conducted a function prediction analysis of the rhizosphere bacteria community in collected HD rhizosphere samples at 50 DPS. The results showed that a total of 15 KEGG level two pathways and 61 KEGG level three pathways were significantly enriched in sandy soil and sandy loam soil compared to black soil and farm soil (Figure 6 and Supplementary Figure 6). We found that in sandy and sandy loam soil, bacteria were highly enriched in amino acid metabolism, carbohydrate metabolism, energy metabolism, membrane transport, etc. These pathways implied that the interaction between rhizosphere bacteria and cucumber roots was stronger in sandy soil and sandy loam soil than in black soil and farm soil. There was a strong exchange of compounds and energy between them. Meanwhile, the pathways of xenobiotic biodegradation and metabolism were also enriched, such as benzoate degradation, aminobenzoate degradation, and polycyclic aromatic hydrocarbon degradation, which showed that bacteria helped degrade substances that were harmful to cucumber growth. Notably, a biofilm formation, ABC transporter, and two-component system (TCS) were the three most enriched pathways with a z-score of 5.1, 7.5, and 7.9, respectively. These three pathways may play the most important role in the process by which bacteria promote plant growth.

We also found other significantly enriched metabolic pathways, such as nitrogen metabolism, pyruvate metabolism, an amino sugar, and nucleotide sugar metabolism. The rapid exchange of carbon and nitrogen to reach a balance between bacteria and plants is also an interesting phenomenon. These abundant carbon metabolisms ensure the survival of bacteria, which, in turn, may promote plant growth by increasing nitrogen absorption and utilization.

## Discussion

In this study, we investigated the comprehensive influence of soil type, genotype, and cultivation time on the rhizosphere bacterial community composition and the structure of cucumbers. Our results illustrate a model that the genotype-specific cucumber could preferentially recruit certain kinds of rhizosphere bacteria from a specific soil environment for its growth promotion, which strongly supports the notion that soil, genotype, and cultivation time of plants, are key interactive factors for the composition and structure of the rhizosphere bacterial community.

Soil is the original source of plant rhizosphere microorganisms. Previous studies have revealed that the microbial community in the soil was significantly affected by soil pH and the C: N ratio (Zarraonaindia et al., 2015). In this study, we found that the bacterial community composition in sandy soil and sandy loam soil was similar. On the contrary, black soil and farm soil showed specific bacterial community structures (Figure 3A); the different physical and chemical characteristics of soil may be one of the reasons. Root exudate was one of the most important factors affecting the composition of plant rhizosphere microorganisms (Huang et al., 2014). Our study showed that different soils shared some common bacterial genera. For example, the rhizosphere of cucumbers grown in four kinds of soil contained *Chloroflexi* and *Firmicutes* at 30 DPS and *Actinobacteriota* at 50 DPS (Figure 3B). We speculated that the similar exudates produced by cucumber roots recruited these bacteria from different soils, and these bacteria played a conservative role in supporting the growth and development of cucumbers. In contrast, the relative abundance of *Firmicutes* and *Gemmatimonadota* varied across these soils at 50 DPS, suggesting that the cucumbers grown in different kinds of soil had reformed the composition of root exudates to recruit certain kinds of bacteria to help themselves adapt to the variable environment. In a study on *Arabidopsis*, significant differences were found in both the rhizosphere exudates produced by plants and the composition of the microbial community at different growth stages (Zhao et al., 2021).

In our study, the beta diversity analysis showed that at the early (30 DPS) and late (50 DPS) stages of cucumber cultivation, the structures of the rhizosphere bacterial community in sandy soil and sandy loam soil were similar, but there was no such trend in black soil and farm soil. Therefore, we speculate that cucumbers had formed a stable rhizosphere bacterial community in sandy soil and sandy loam soil at an early stage, while in black soil and farm soil, cucumbers needed longer to form such a stable bacterial community. Therefore, this phenomenon might be caused by the joint effects of the soil type and plant cultivation time. A similar phenomenon was also found in wheat; the rhizosphere microbial community was relatively stable during the seedling, tillering, and flowering stages; however, significant microbial structure changes were observed during the mature stage (Ma et al., 2021).

We found that wild-type cucumbers had better recruitability to certain bacteria than cultivated lines. The result of 16S rRNA sequencing showed that HD could recruit more *Nitrospira*, *Nocardioides, Gaiella*, *Bacillus*, *Tumebacillus*, and *Paenibacillus* for sandy soil and sandy loam soil (Figure 4). At the same time, KEGG pathway enrichment analysis showed that nitrogen metabolism and tryptophan metabolism (precursor substance of IAA) (Islam et al., 2016) related pathways were significantly enriched (Figure 6). *Nitrospira* plays an essential role in the global nitrogen cycle, which can oxidize nitrite to nitrate and degrade ammonium. Studies showed that *Nitrospira* played a vital role in the process of the soil nitrogen cycle, especially in low-N conditions (Attard et al., 2010). It was also found that *Nitrospira* was one of the top bacterial genera recruited by the rice root and was related to nitrogen absorption and utilization (Zhang J. et al., 2019). Therefore, *Nitrospira* may play an important role in promoting nitrogen absorption and utilization in cucumbers, although the mechanisms are still unclear. *Paenibacillus* is a well-studied PGPR that can colonize the root surface of cucumbers and effectively improve the yield of cucumbers by fixing more nitrogen from the environment (Li et al., 2008; Hao and Chen, 2017). *Nocardioides* could produce a large amount of IAA to help wheat resist salt stress (Meena et al., 2020). In addition, in a drought environment, *Nocardioides* helped nitrogen recycle through leucine aminopeptidase and chitinase activities (Zhang X. et al., 2021). Studies have indicated that *Gaiella* played an important role in nitrogen cycling in cultivated farm ecosystems (Zhang B. et al., 2019). *Bacillus* is one of the most important PGPR that promotes plant growth and development by secreting phytohormones (Shahzad et al., 2021), improving nutrient absorption and utilization efficiency (Sun et al., 2021) and stress tolerance (Shahid et al., 2021). Park et al. (2013) showed that *B*. *subtilis* strain B4 increased IAA activity by increasing L-tryptophan metabolism to encourage cucumber growth. *Tumebacillus* was first reported in 2008 (Steven et al., 2008), but until now, its relationship with plant growth promotion remains unclear.

In addition to the nitrogen metabolism and tryptophan metabolism pathways, biofilm formations, ABC transporters, and TCS-related pathways were also enriched. Biofilm formation is key to the successful root colonization of soil bacteria (Knights et al., 2021). ABC transporters in bacteria can absorb substrates, such as sugars, amino acids, metal ions, iron chelating agents, and vitamin B-12, to support their survival. In addition, it is involved in the efflux of bacterial substances, such as bacterial surface components (lipopolysaccharides, capsular polysaccharides) and siderophores (Davidson and Chen, 2004). The TCS is a ubiquitous mechanism for bacteria in nature to respond to environmental stimuli (Capra and Laub, 2012). This implied that the interaction between rhizosphere bacteria and cucumber roots was stronger in sandy soil and sandy loam soil than in black soil and farm soil. There was an active exchange of compounds and energy between them by ABC transporters and TCS.

We also observed that flagellar assembly and bacterial chemotaxis-related pathways were significantly enriched in the rhizosphere bacteria of HD, which could help the bacteria move quickly toward the plant roots when sensing exudates (Arnaouteli et al., 2021). Interestingly, we also found the enrichment of the phosphotransferase system (PTS) pathway. Researchers discovered its carbon phosphorylation function as early as 1964. Over the past several decades, additional functions of PTS have been revealed, such as its role in sugar transport and phosphorylation, nitrogen and phosphorus metabolism, and bacterial biofilm formation (Saier, 2015). At the same time, the pathways related to carbon source metabolisms, such as pyruvate, fructose, and mannose metabolism, were notably enriched (Figure 6). Studies showed that exudates can mediate *Bacillus* root colonization and biofilm formation in cucumbers and that effective root colonization is a prerequisite for growth promotion (Bhatia et al., 2021). In turn, cucumber provided a large amount of carbon source for bacteria, which the PTS system could utilize to support its growth. In *B*. *velezensis* SQR9, TCS was used to break through the immune system of plants and enhance their root colonization (Zhang H. et al., 2021). Besides, we also found an enrichment pathway of siderophore biosynthesis (Supplementary Figure 6). Siderophore is a kind of iron chelator, which PGPR can use to acquire iron from the soil to support the growth of itself and its host plant (Radzki et al., 2013). Therefore, according to the above analysis, we propose a model for the interaction mechanism between cucumbers and the rhizosphere bacteria. We assumed that after bacteria perceive the recruitment signal of the cucumber, they approach the root by chemotaxis and flagellar movement and form a robust biofilm on the surface of the root by TCS and PTS. After that, using the ABC transfer system, they can acquire the nutrients, discharge the metabolites, and use the PTS to digest these nutrients compounds. Finally, some of these metabolites, such as IAA, nitrogen sources, and siderophores, were administered to cucumbers to promote growth and development.

In conclusion, our study showed that soil type, genotype, and cultivation time can collectively affect the rhizosphere bacterial community composition and the structure of cucumbers. Meanwhile, the wild-type cucumber HD could recruit specific rhizosphere bacteria from sandy soil and sandy loam soil, including *Nitrospira*, *Nocardioides*, *Bacillus, Tumebacillus*, and *Paenibacillus*, compared to cultivated cucumber XT to support its better growth. According to the result of functional prediction of the rhizosphere bacterial community, we found that the rhizosphere of HD was enriched in pathways related to nitrogen metabolism, biofilm formation, ABC transporters, and PTS and TCS, which were mainly used for bacterial metabolism and exchanging substances and energy with the root system. Our findings suggest that the wild-type cucumber has better environmental adaptability due to its stronger rhizosphere bacteria recruitment capacity. In future studies, it will also be interesting to reveal the recruitment mechanism of the wild-type cucumber to specific bacteria and how these bacteria promote plant growth.

## Data Availability Statement

The datasets presented in this study can be found in online repositories. The names of the repository/repositories and accession number(s) can be found below: https://ngdc.cncb.ac.cn/search/?dbId=&q=PRJCA008005, PRJCA008005.

## Author Contributions

YQ, XY, and SH designed the study. ZL, YZ, and YQ performed the experiments and analyzed the 16S rRNA sequencing data. YL provided technical support to this study. ZL, YZ, YQ, and XY wrote the manuscript. SH provided critical comments and edited the manuscript. All authors contributed to the article and approved the submitted version.

## Conflict of Interest

The authors declare that the research was conducted in the absence of any commercial or financial relationships that could be construed as a potential conflict of interest.

## Publisher’s Note

All claims expressed in this article are solely those of the authors and do not necessarily represent those of their affiliated organizations, or those of the publisher, the editors and the reviewers. Any product that may be evaluated in this article, or claim that may be made by its manufacturer, is not guaranteed or endorsed by the publisher.

## Footnotes

1
http://fao.org

## Supplementary Material

Supplementary Figure 1 Relative abundance of top 20 of HD rhizosphere of four kinds of soils at 50 DPS. Blue is sandy soil; purple is sandy loam soil, green is black soil, and black is farm soil. Data are the means of three replicates, and error bars indicate standard deviations.

Supplementary Figure 2 Relative abundance of top 20 of HD rhizosphere in sandy soil at 30 and 50 DPS. Purple is 30 DPS; blue is 50 DPS. Data are the means of three replicates, and error bars indicate standard deviations.

Supplementary Figure 3 Relative abundance of top 20 of XT and HD rhizosphere in sandy soil at 50 DPS. Purple is HD; blue is XT. Data are the means of three replicates, and error bars indicate standard deviations.

Supplementary Figure 4 Relative abundance of top 20 of HD rhizosphere in sandy loam soil at 30 and 50 DPS. Purple is 30 DPS; blue is 50 DPS. Data are the means of three replicates, and error bars indicate standard deviations.

Supplementary Figure 5 Relative abundance of top 20 of XT and HD rhizosphere in sandy loam soil at 50 DPS. Purple is HD; blue is XT. Data are the means of three replicates, and error bars indicate standard deviations.

Supplementary Figure 6 KEGG pathways of rhizosphere bacteria sample in different soils. Pathways are enriched in sandy and loam soil compared to black soil and farm soil. Pathways with a significant difference in reporter score (>1.64, enriched in sandy soil and sandy loam soil) were retained. Reporter scores >1.64 are shown on the map. Different colors represent different metabolic pathways of KEGG level 2 **(right)** and level 3 **(left)**.
