# Supplementary figures and images for: Genotype-Specific Recruitment of Rhizosphere Bacteria From Sandy Loam Soil for Growth Promotion of Cucumis sativus var. hardwickii
Source: Front Microbiol. 2022 Jun 27;13:910644. doi: 10.3389/fmicb.2022.910644 (PMC9271904; doi:10.3389/fmicb.2022.910644)

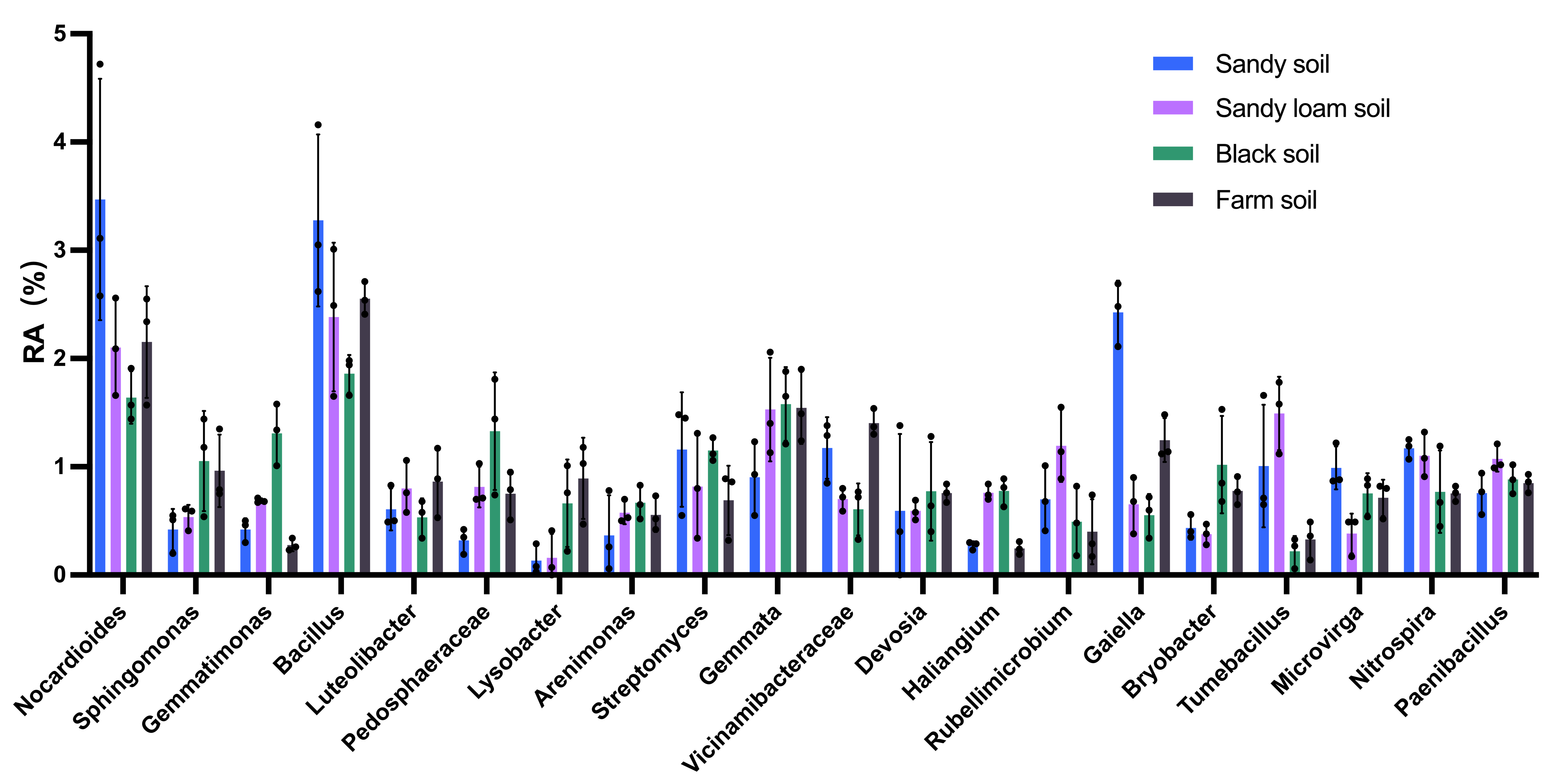

Supplement: Supplementary Figure 1 — Relative abundance of top 20 of HD rhizosphere of four kinds of soils at 50 DPS. Blue is sandy soil; purple is sandy loam soil, green is black soil, and black is farm soil. Data are the means of three replicates, and error bars indicate standard deviations. [file Image_1.TIFF]

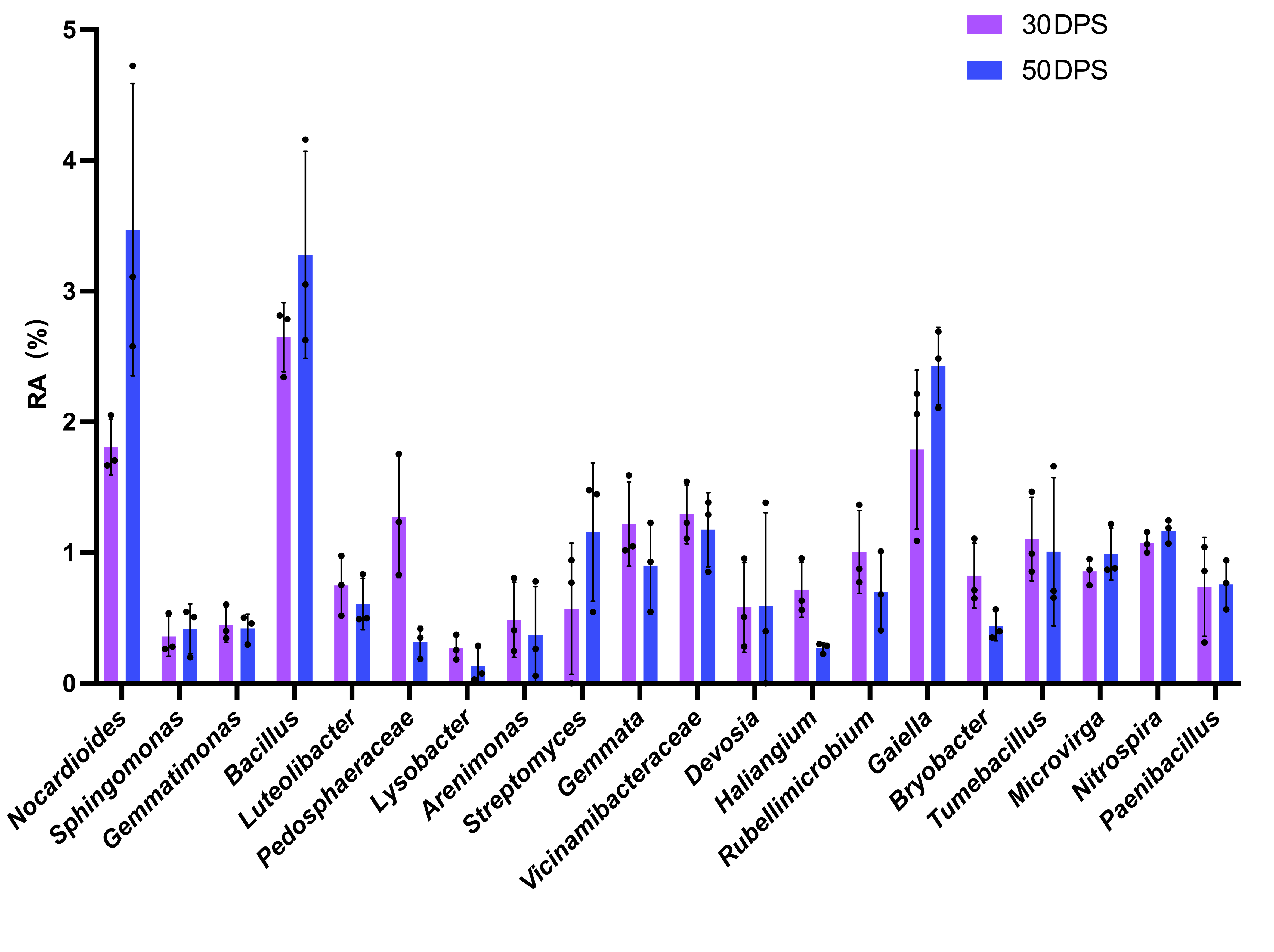

Supplement: Supplementary Figure 2 — Relative abundance of top 20 of HD rhizosphere in sandy soil at 30 and 50 DPS. Purple is 30 DPS; blue is 50 DPS. Data are the means of three replicates, and error bars indicate standard deviations. [file Image_2.TIFF]

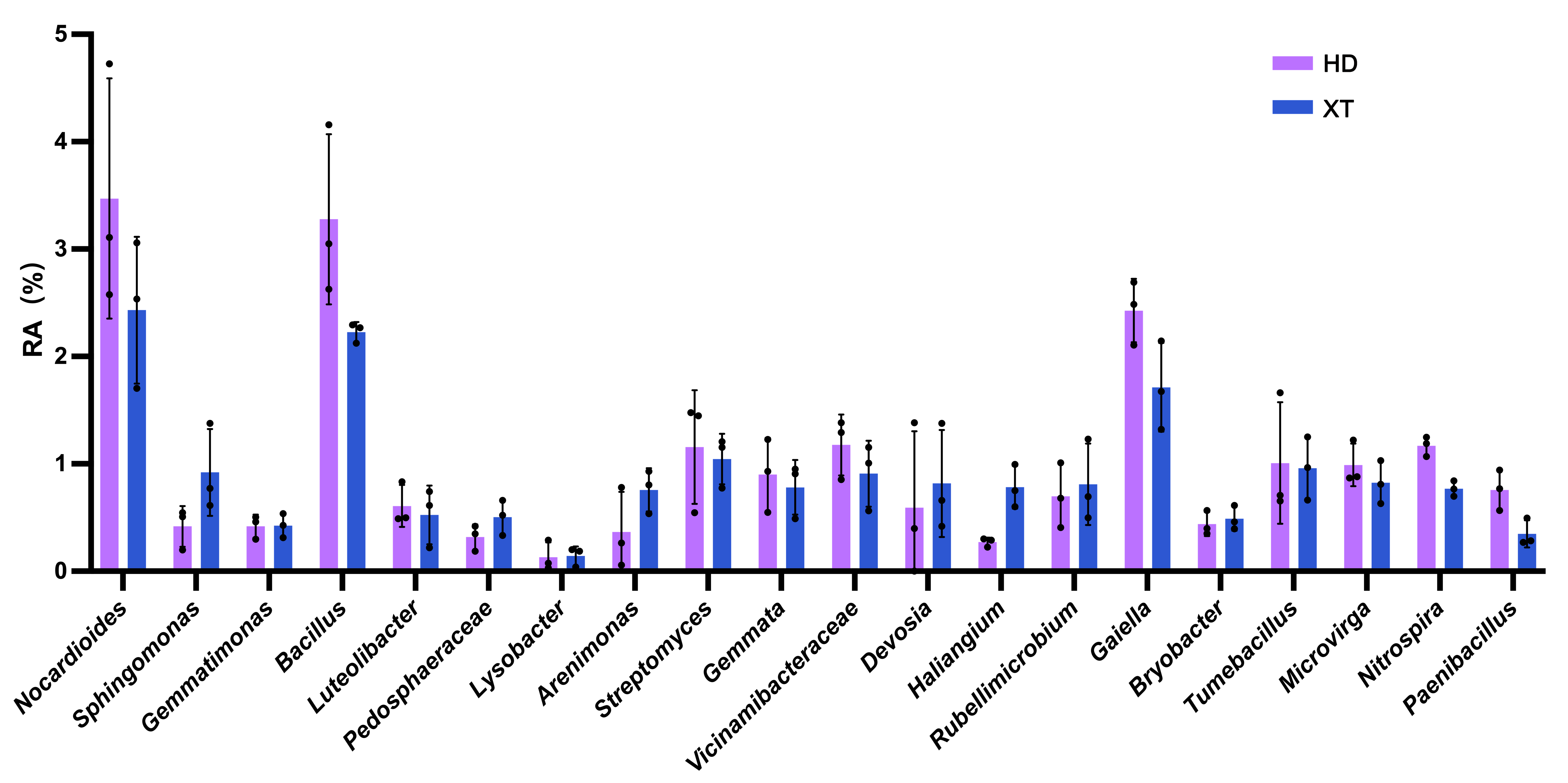

Supplement: Supplementary Figure 3 — Relative abundance of top 20 of XT and HD rhizosphere in sandy soil at 50 DPS. Purple is HD; blue is XT. Data are the means of three replicates, and error bars indicate standard deviations. [file Image_3.TIFF]

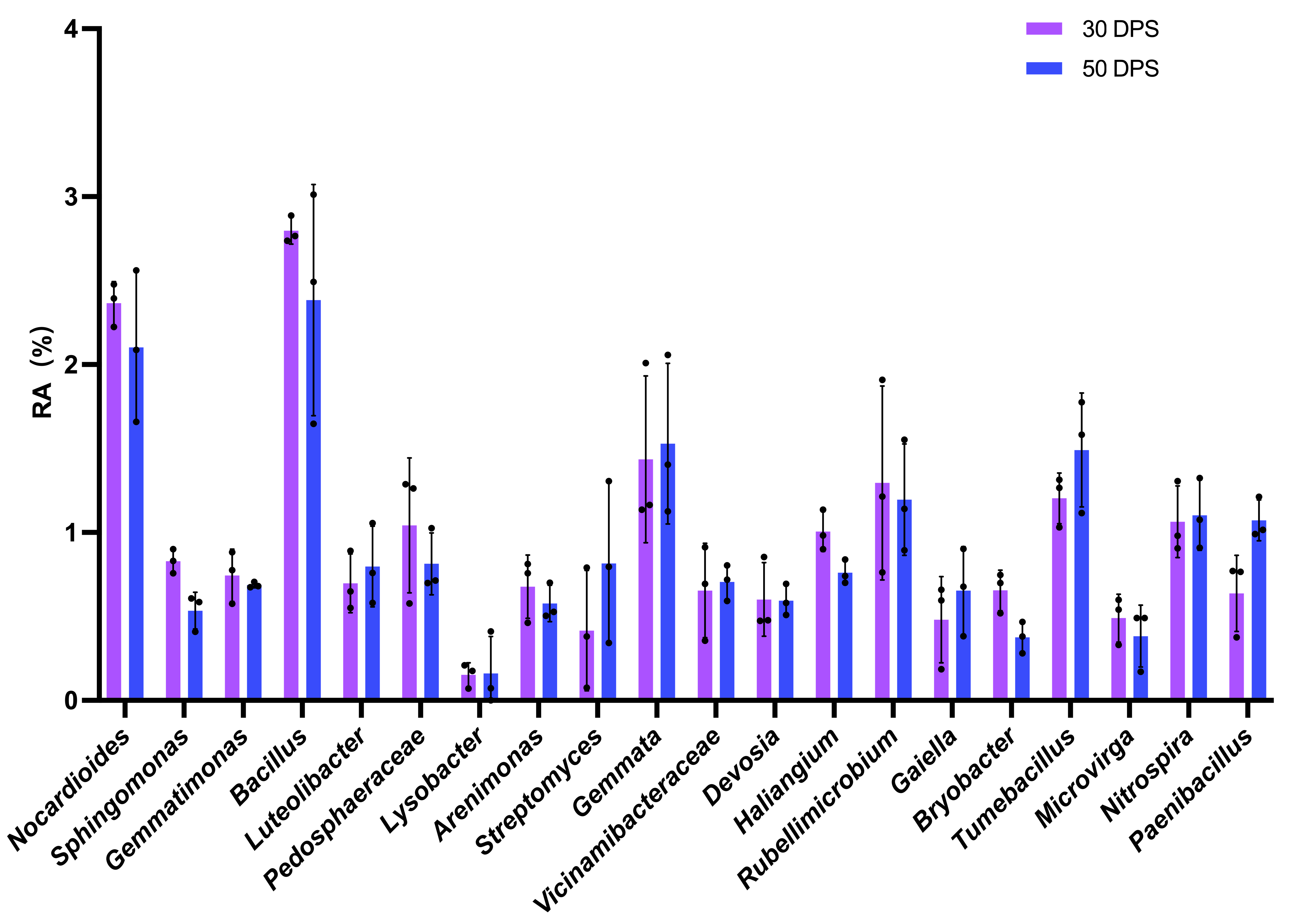

Supplement: Supplementary Figure 4 — Relative abundance of top 20 of HD rhizosphere in sandy loam soil at 30 and 50 DPS. Purple is 30 DPS; blue is 50 DPS. Data are the means of three replicates, and error bars indicate standard deviations. [file Image_4.TIFF]

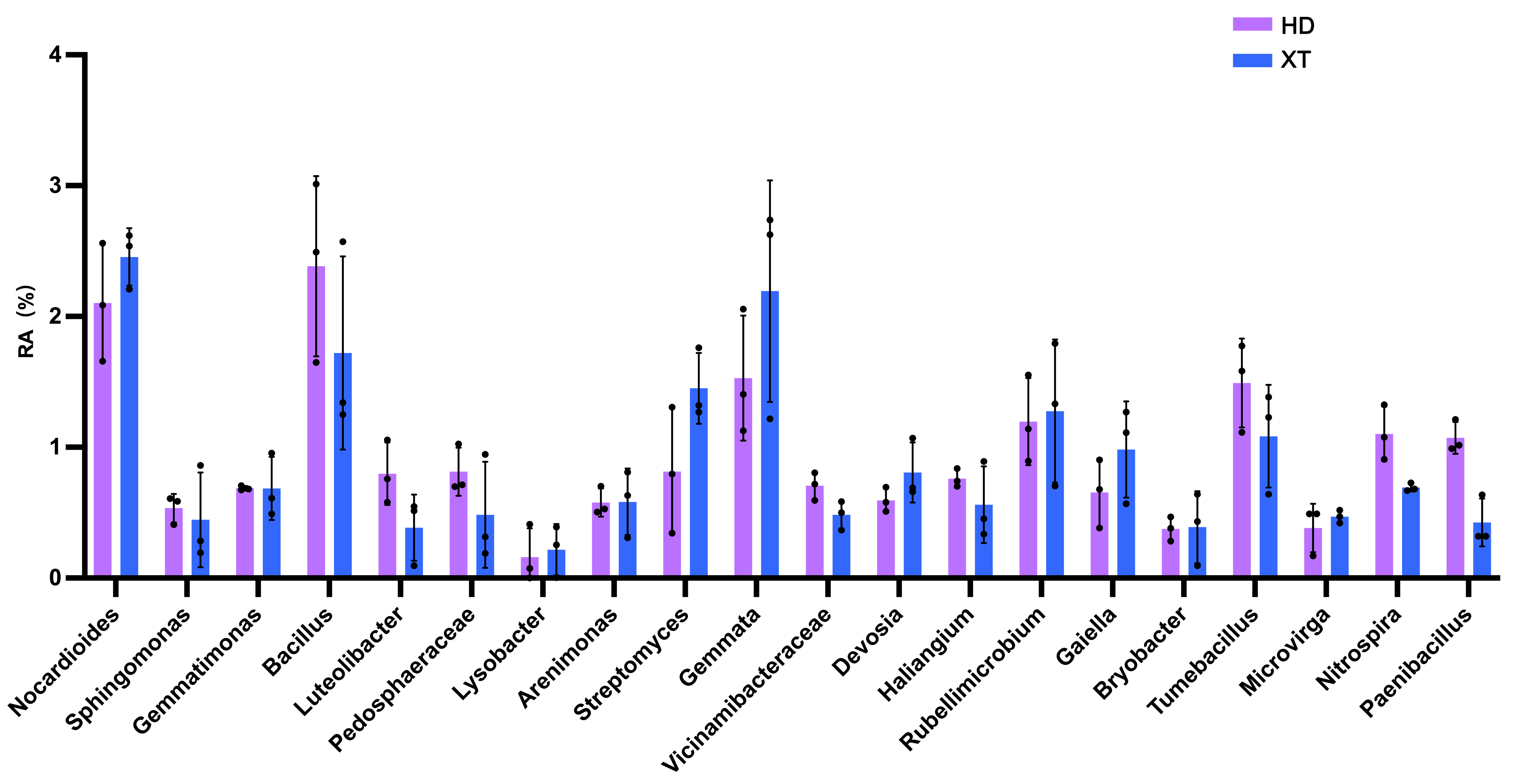

Supplement: Supplementary Figure 5 — Relative abundance of top 20 of XT and HD rhizosphere in sandy loam soil at 50 DPS. Purple is HD; blue is XT. Data are the means of three replicates, and error bars indicate standard deviations. [file Image_5.TIFF]

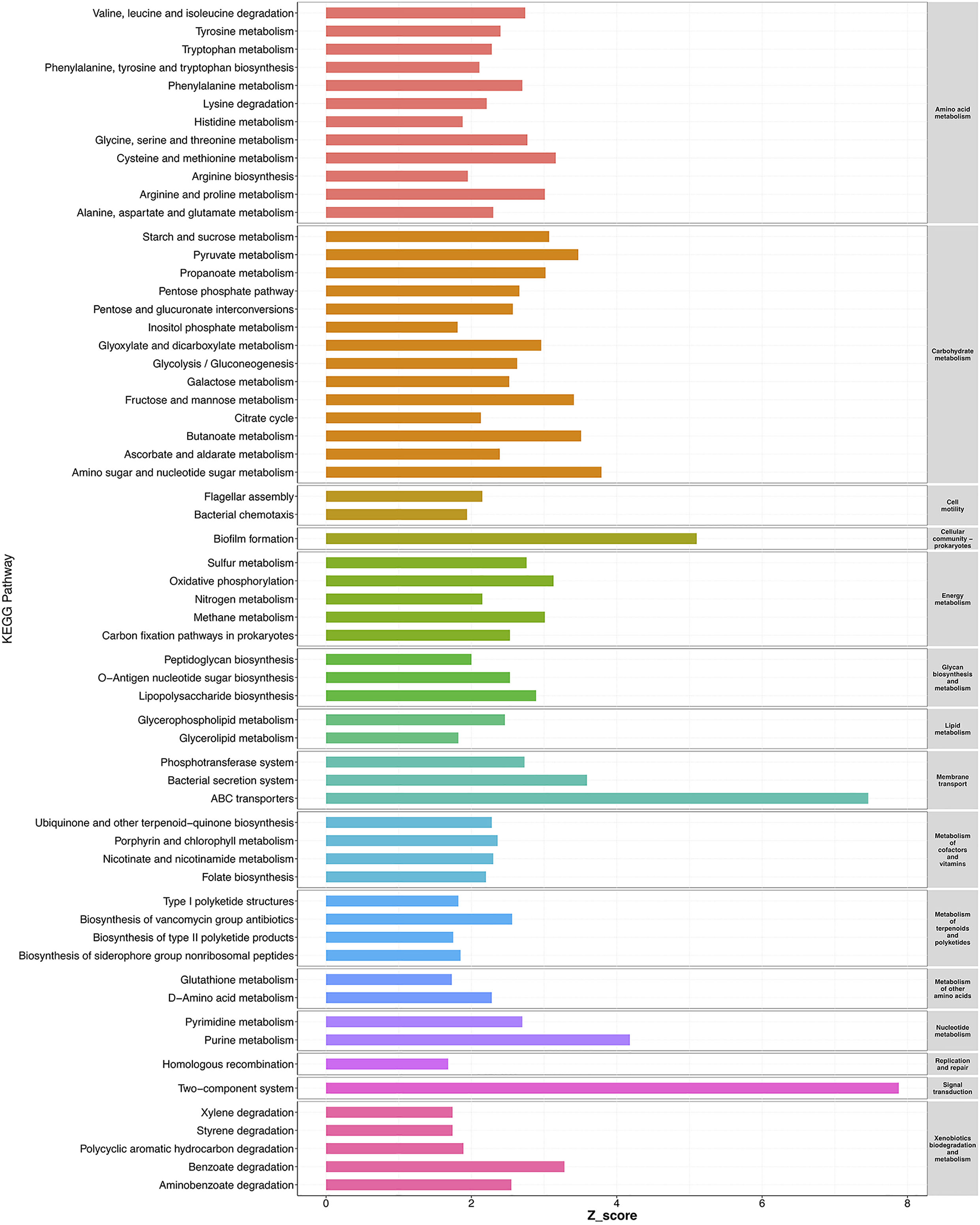

Supplement: Supplementary Figure 6 — KEGG pathways of rhizosphere bacteria sample in different soils. Pathways are enriched in sandy and loam soil compared to black soil and farm soil. Pathways with a significant difference in reporter score (>1.64, enriched in sandy soil and sandy loam soil) were retained. Reporter scores >1.64 are shown on the map. Different colors represent different metabolic pathways of KEGG level 2 (right) and level 3 (left). [file Image_6.JPEG]
